# The efficacy and safety of tivantinib in the treatment of solid tumors: a systematic review and meta-analysis

**DOI:** 10.18632/oncotarget.22615

**Published:** 2017-11-03

**Authors:** Hao Zhang, Zhengqiang Bao, Hongwei Liao, Wen Li, Zhihua Chen, Huahao Shen, Songmin Ying

**Affiliations:** ^1^ Department of Respiratory and Critical Care Medicine, Second Affiliated Hospital, Institute of Respiratory Diseases, Zhejiang University School of Medicine, Hangzhou, China; ^2^ Department of Cancer Center, The Second Hospital of Shandong University, Jinan, China; ^3^ Department of Pharmacology, Zhejiang University School of Medicine, Hangzhou, China

**Keywords:** tivantinib, ARQ197, MET, NSCLC, hepatocellular carcinoma

## Abstract

**Background:**

Tivantinib was designed to kill cancers by targeting the mesenchymal-epithelial transition (MET) protein. Although numerous tivantinib clinical trials are ongoing, tivantinib's efficacy and safety are still not clear. This meta-analysis was done to evaluate tivantinib's efficacy and safety in solid tumor treatment.

**Materials and Methods:**

PUBMED, EMBASE, and other databases were searched for eligible tivantinib clinical trials. The hazard ratio (HR) and 95% confidence interval (CI) of progression-free and overall survival (PFS and OS, respectively) were pooled and analyzed to evaluate tivantinib's efficacy. Data concerning adverse events (Grade ≥ 3) were pooled to calculate relative risks (RRs) with 95% CI for tivantinib-treated compared with control arms.

**Findings:**

Patients (1824) from six randomized control trials (RCTs) were enrolled. Compared with controls, tivantinib produced a significant improvement in PFS (HR, 0.73; 95% CI 0.65–0.83) but not in OS. In the non-small-cell lung cancer (NSCLC) subgroup, tivantinib combined with erlotinib prolonged patients' PFS when compared with controls (HR, 0.75; 95% CI, 0.65–0.86). In the white population, tivantinib also significantly improve PFS between treatment and control arms (HR, 0.75; 95% CI, 0.65–0.87). Tivantinib significantly improved OS in patients with high levels of MET expression. Tivantinib was shown to increase the risk of anemia and neutropenia.

**Interpretation:**

Tivantinib was better in prolonging PFS (not OS) in patients with solid tumors. High MET expression cancers may benefit from tivantinib. Tivantinib appeared to be well-tolerated by patients.

## INTRODUCTION

The mesenchymal-epithelial transition (MET) protein is a member of the tyrosine kinase receptor superfamily. It is encoded by the MET proto-oncogene. Hepatocyte growth factor (HGF) has been shown to be the ligand of MET. The MET/hepatocye growth factor pathway appears to have important roles in tumor proliferation and invasion [1]. Normally, MET appears to be widely expressed in very low levels in all kinds of tissues. However, it has been shown to often be aberrantly activated in solid tumors. Previous studies have suggested that MET was overexpressed or amplified in various human cancers, especially in non-small cell lung cancer (NSCLC) [2–4]. Patients overexpressing MET may have poor clinical outcomes. It was reported that median disease-free survival in patients with low levels of MET expression was as long as 53 months; in contrast, however, it was only eight months in patients with high levels of MET expression [5]. Approximately one fifth of epidermal growth factor receptor (EGFR) inhibitor resistance in NSCLC was related to MET amplification [6]. MET amplification may also contribute to tumor metastasis as MET expression was significantly higher in metastatic cancers than primary cancers [7]. Thus, targeting MET may be an effective strategy for cancer therapy. Until now, numerous molecular inhibitors have been designed to treat cancers via inhibition of MET activity. These inhibitiors include cabozantinib, amuvatinib, criotinib, and foretinib in addition to others. However, most of these inhibitors are multi-target drugs, targeting not only MET but also anaplastic lymphoma kinase (ALK), AXL, VEGFR2, RET, and KIT [8]. Although some of them produced good clinical outcomes, we couldn't determine whether it was due to MET or other target suppression.

Tivantinib (AQR197) is a highly selective MET inhibitor. It can inhibit MET phosphorylation and downstream signaling pathways [9]. It has been reported in various cancer cell lines that tivantinib can bind MET in non-phosphorylated or inactive form, inhibit both constitutive and ligand-mediated MET autophosphorylation, then maintain this inactive state [10]. Not like other ATP dependent C-Met kinase inhibitors, tivantinib was non-ATP competitive. Besides, it inhibited c-Met with an inhibitory constant[Ki] of only 355 nmol/L, which suggested it was not potent enough to inhibit other human kinases, even Ron kinase which also belongs to the same family of c-Met [9].

In recent years, many clinical trials have been done or are ongoing in order to evaluate tivantinib's effects against various solid tumors such as NSCLC, hepatocellular carcinoma, and colorectal, prostate, and gastric cancers in addition to others. Some of the trials have been beneficial to patients. For example, erlotinib was one of first generation tyrosine kinase inhibitors, and while it performed well in the treatment of EGFR mutation lung cancers, most of patients would eventually present resistance to erlotinib. However, when combined with tivantinib, erlotinib appeared to significantly prolong patients’ progression-free survival (PFS) [11]. In a phase II trial, tivantinib failed to meet prespecified statistical targets for efficacy since the overall response rate was only 5% [12]. Thus, tivantinib's efficacy in the treatment of solid tumors seems questionable. In addition, as with other anti-tumor agents, tivantinib was shown to induce some adverse events (AEs) such as interstitial lung disease, anemia, fatigue, neutropenia, and leukopenia [13]. In this meta-analysis, we aimed to explore the efficacy and safety of tivantinib in the treatment of solid tumors.

## RESULTS

### Literature search

**Figure 1:**
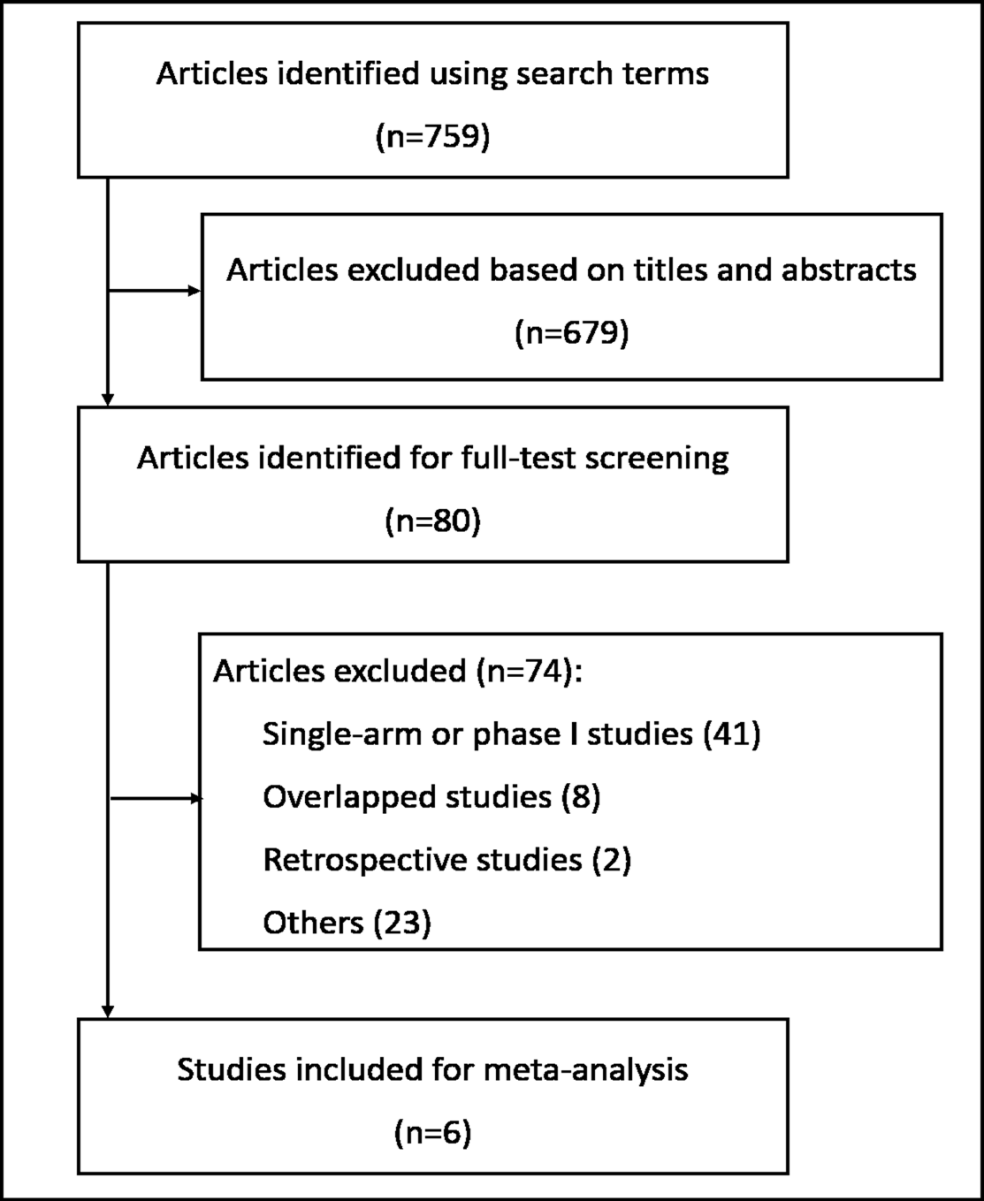
Flow diagram of the literature search and trial selection process

According to our retrieval strategy, a total of 759 items were identified. After the first round of screening by title and abstract, 679 irrelevant articles were excluded, and 80 articles remained for full review. After carefully reviewing the full texts, 74 articles were excluded: 1.) 41 were single arm or phase I studies; 2.) eight were overlapping studies; 3.) two were retrospective studies; and 4) 23 articles contained no relative outcomes. Ultimately, six eligible RCTs were included for analysis (Figure 1). Among the six included articles, three were NSCLC-related and one was related to hepatocellular carcinoma, one to colorectal cancer, and one to prostate cancer [11, 13, 14–17]. Data from a total of 1824 patients were included. Detailed information is presented in Table 1.

**Table 1:** Characteristics of the included randomized controlled trials

Study	Year	Phase	Histology	Cases	Median age (Range),y	Female NO. (%)	Ethnicity,No. (%)	Met-high No. (%)	Control	Treatment
Scagliotti	2015	III	NSCLC	1048	61.5 (24-89)	429 (40.9)	White 876 (83.6)	211 (20.1)	Placebo plus Erlotinib	Tivantinib plus Erlotinib
Sequist	2011	II	NSCLC	167	63 (23-89)	67 (40.1)	White 158 (94.6)	37 (22.2)	Placebo plus Erlotinib	Tivantinib plus Erlotinib
Yoshioka	2015	III	NSCLC	307	63 (27-84)	96 (31.3)	Asian (100)	160 (52.1)	Placebo plus Erlotinib	Tivantinib plus Erlotinib
Eng	2016	I/II	Colorectal Cancer	117	57 (27-79)	59 (50)	Caucasian 111(95)	44 (38)	Placebo plus CETIRI	Tivantinib plus CETIRI
Santoro	2013	II	HC	107	69 (27-85)	21 (19.6)	White 96 (89.7)	37 (34.6)	Placebo	Tivantinib
Monk	2015	II	Prostate Cancer	78	67 (43-85)	0 (0)	NR	NR	Placebo	Tivantinib

NSCLC: Non-small-cell-lung-cancer. HC: Hepatocellular carcinoma. CETIRI: Cetuximab+Irinotecan. NR: Not reported.

### PFS and OS

**Figure 2:**
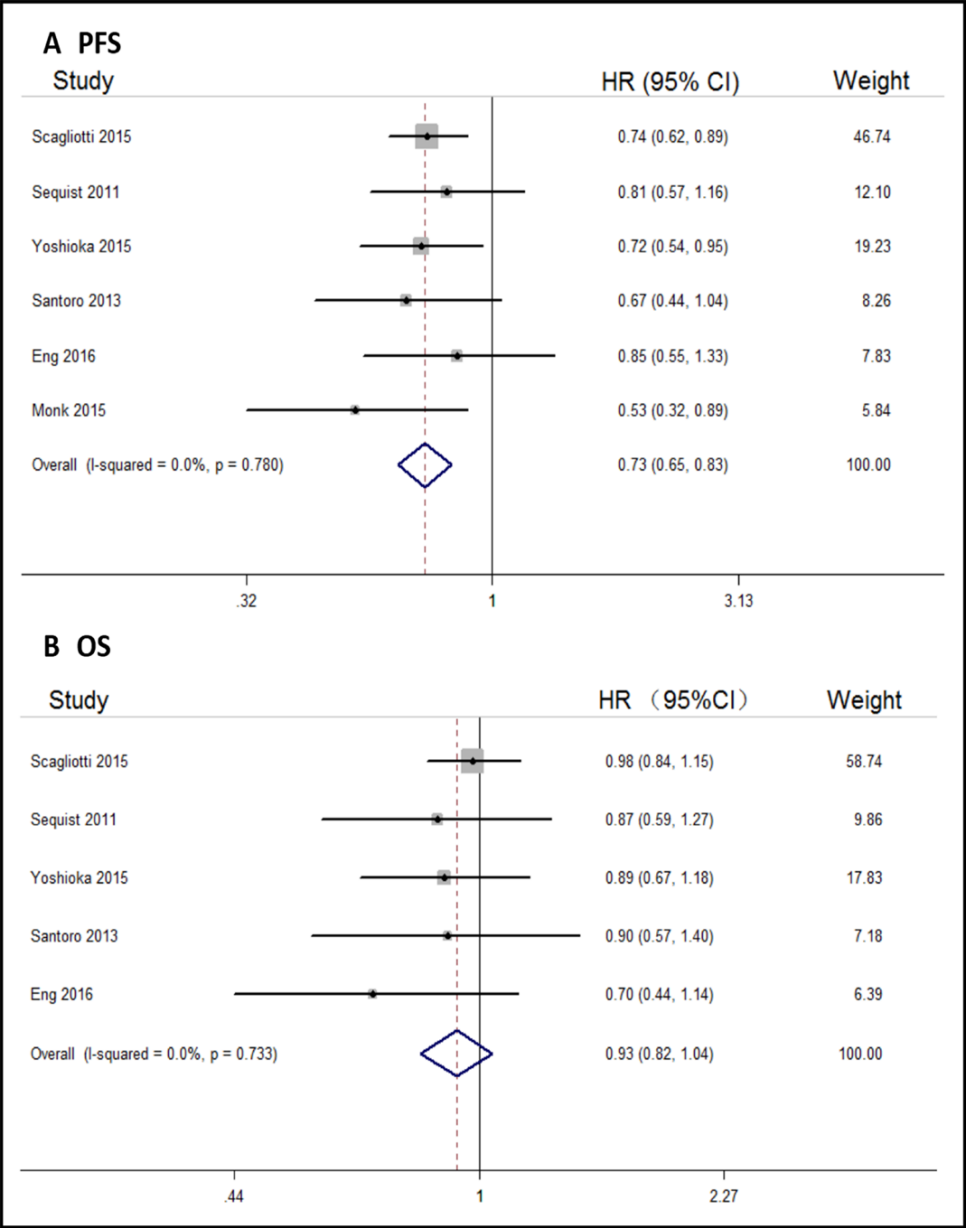
Forest plots of the pooled HRs for PFS and OS by overall population (**A**) PFS, (**B**) OS.

All the six trials reported PFS for the cancer study population. Compared with control arms, tivantinib could significantly prolong PFS in solid tumor patients (HR, 0.73; 95% CI, 0.65–0.83; Figure 2A). Five of the six trials reported OS of the cancer study population, and there were no differences in OS between tivantinib and control arms (HR, 0.93; 95% CI, 0.82–1.04; Figure 2B).

### Subgroup analysis

**Figure 3:**
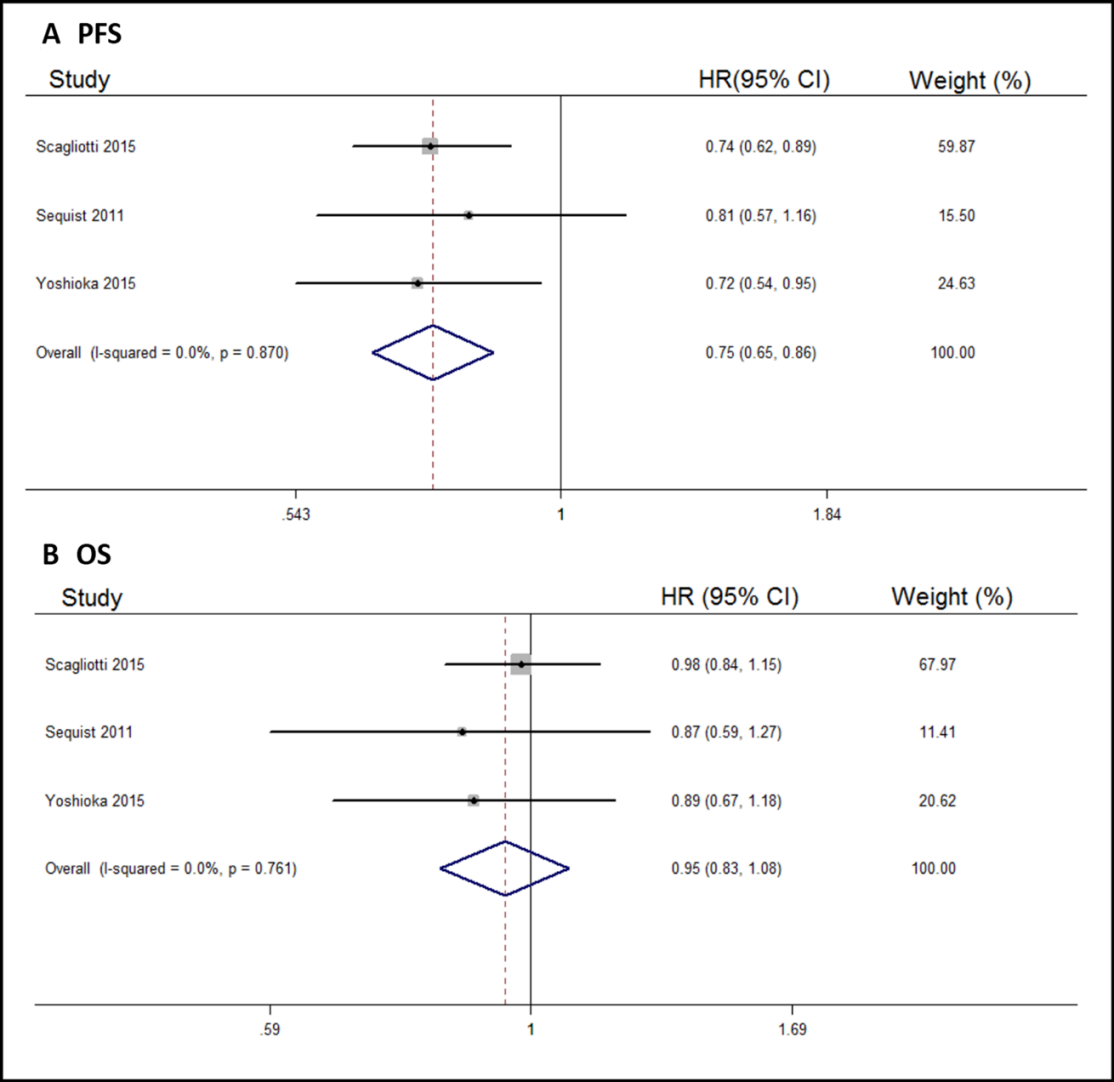
Forest plots of the pooled HRs for PFS and OS by NSCLC subgroup (**A**) PFS, (**B**) OS.

Three of the six included trials described NSCLC treatment, so we carried out a subgroup analysis to assess the efficacy of tivantinib for NSCLC treatment. Compared with control arms, tivantinib did well in prolonging the PFS of NSCLC patients (HR, 0.75; 95% CI, 0.65–0.86; Figure 3A). However, OS improvement was not significant (HR, 0.95; 95% CI, 0.83–1.08; Figure 3B).

**Figure 4:**
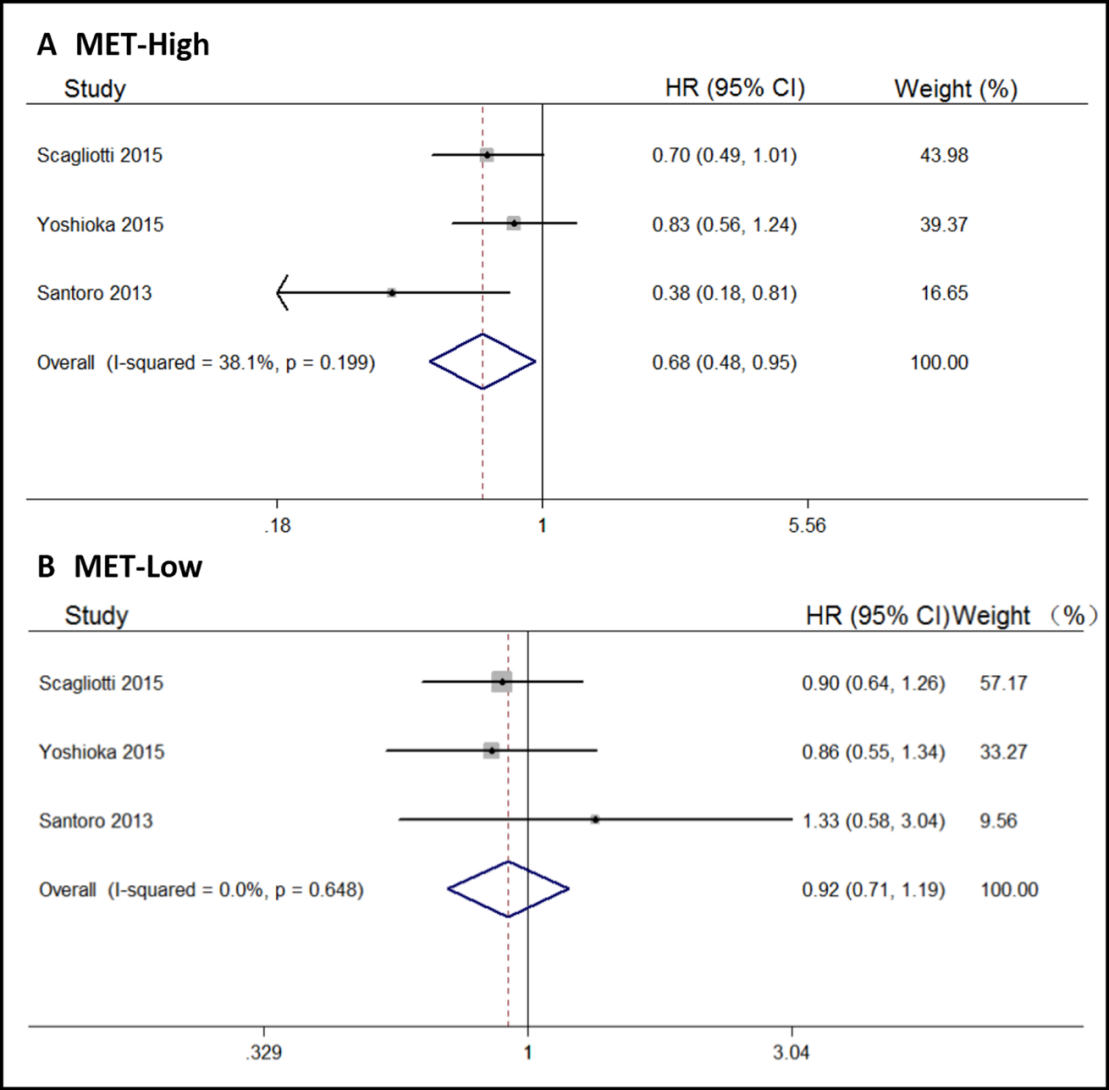
Forest plots of the pooled HRs for OS by MET status subgroup (**A**) MET high subgroup, (**B**) MET low subgroup.

We also analyzed the pooled OS according to MET gene status. According to the included trials, the status of c-MET expression was assessed by immunohistochemistry. When the ≥ 50% of tumor cells showed moderate or strong staining intensity were graded as “c-Met high”, and all remaining samples were graded as “c-Met low”. By our results, tivantinib could significantly prolong OS in patients with high levels of MET expression (HR, 0.68; 95% CI, 0.48–0.95; Figure 4A); in low MET expression patients, there were no differences between tivantinib and control arms (HR, 0.92; 95% CI, 0.71–1.19; Figure 4B).

**Figure 5:**
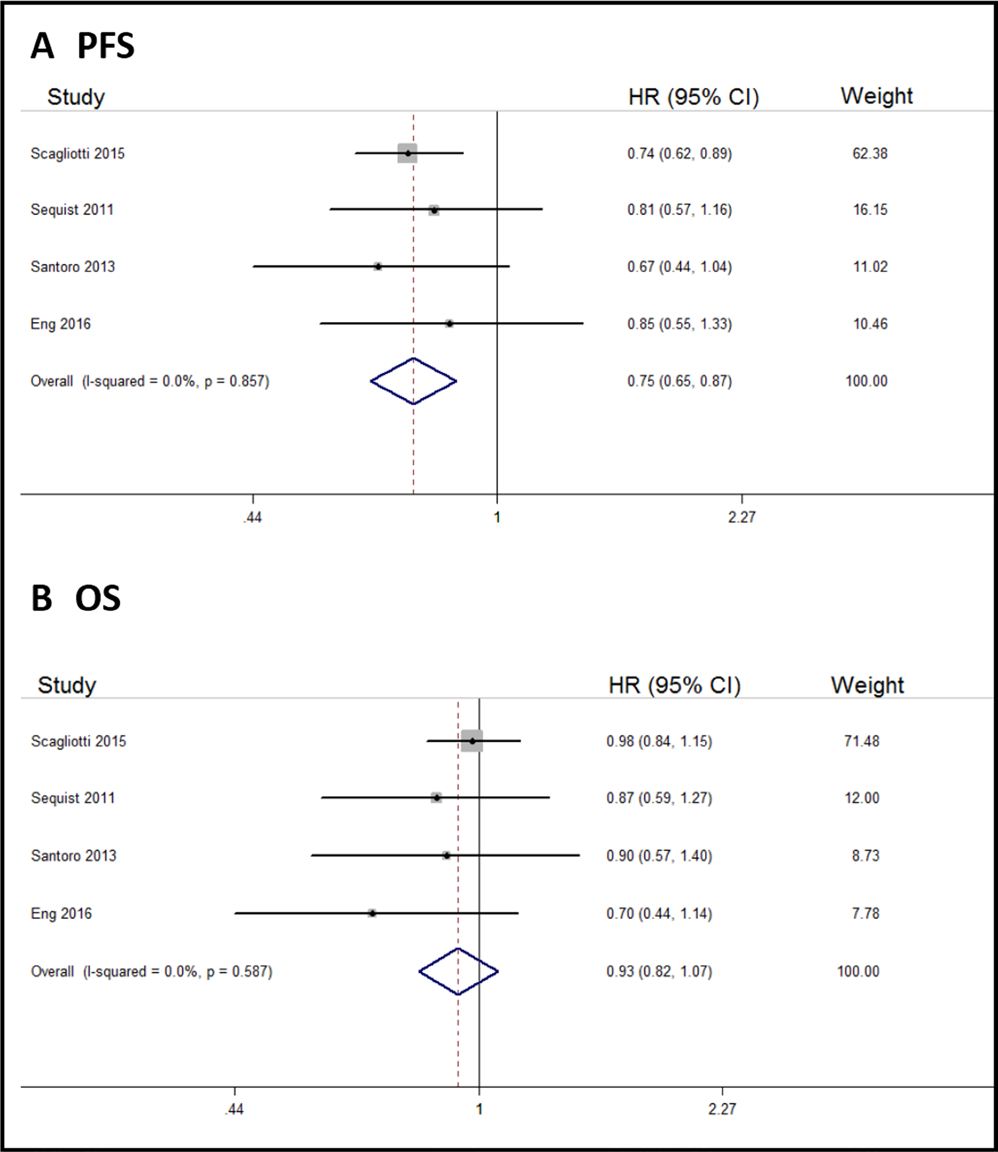
Forest plots of the pooled HRs for PFS and OS by white population subgroup (**A**) PFS, (**B**) OS.

Almost all participants in the four trials were white, so the subgroup analysis was done to assess tivantinib's efficacy in the white population. According to our results, tivantinib could significantly improve white cancer patients’ PFS (HR, 0.75; 95% CI, 0.65–0.87) but not OS (HR, 0.93; 95% CI, 0.82–1.07; Figure 5).

**Figure 6:**
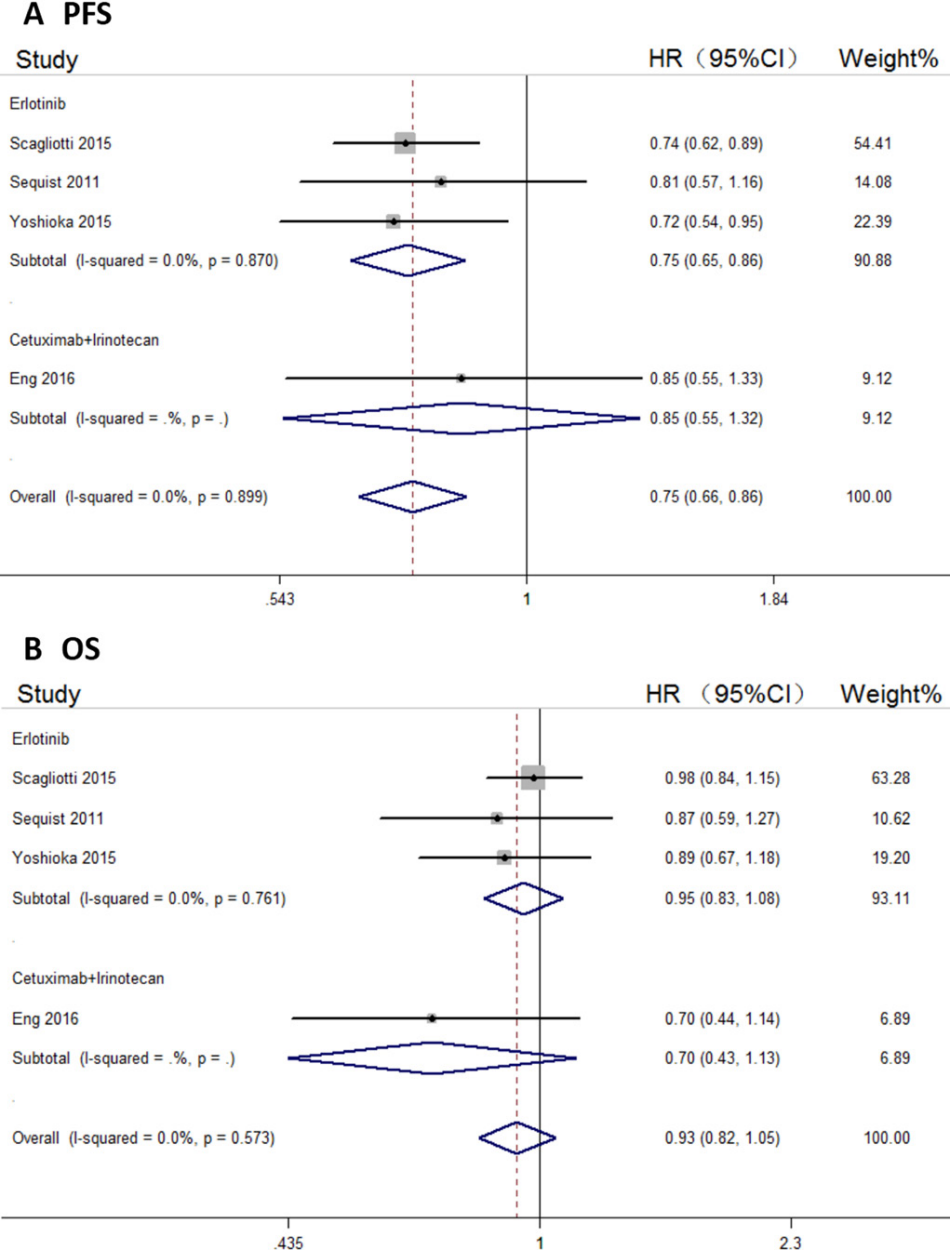
Forest plots of the pooled HRs for PFS and OS by tivantinib combined with erlotinib or Cetuximab+Irinotecan (CETRI) subgroup (**A**) PFS, (**B**) OS.

In four of the six trials, tivantinib was combined with erlotinib or cetuximab+irinotecan (CETIRI). We evaluated the effects of tivantinib addition to other anti-tumor agents and compared this with placebo and erlotinib or CETIRI arms. Tivantinib in combination with erlotinib or CETIRI was shown to significantly improve PFS of cancer patients (HR, 0.75; 95% CI, 0.66–0.86), especially in groups combined with erlotinib (HR, 0.75; 95% CI, 0.65–0.86); there were no differences between tivantinib in combination with CETIRI or placebo in combination with CETIRI (HR, 0.85; 95% CI, 0.55–1.32; Figure 6A). Tivantinib in combination with either erlotinib or CETIRI failed to improve cancer patients’ OS (Figure 6B).

### Safety

To evaluate the safety of tivantinib, we analyzed the pooled AE data with a severity of ≥ Grade 3. The dose of tivantinib was reported as 360 mg twice daily (BID) in all six trials. According to our analysis, the most common AEs were anemia, nausea, neutropenia, and rash. Tivantinib could increase risk of anemia (RR, 2.15; 95% CI, 1.10–4.18) and neutropenia (RR, 5.31; 95% CI, 1.00–28.25). Although tivantinib had a tendency to increase or decrease the risk of other AEs, there were no significant differences between tivantinib and control arms. (Table 2).

**Table 2:** Relative risks with 95% confidence intervals for common adverse events (Grade ≥ 3)

Adverse event	No.of Trials	Subjects	RR [95% CI]	*P*	I^2^	*P*^b^
Anaemia	5	828/818	2.15 [1.10, 4.18]	0.02	33%	0.2
Anorexia	2	208/206	1.27 [0.35, 4.61]	0.72	0%	0.36
Decreased appetite	3	620/612	0.99 [0.51, 1.92]	0.97	0%	1
Dehydration	2	146/142	1.68 [0.45, 6.29]	0.44	0%	0.45
Diarrhoea	3	704/695	0.87 [0.52, 1.44]	0.59	0%	0.57
Dyspnea	2	604/600	0.91 [0.43,1.92]	0.8	57%	0.13
Fatigue	3	246/242	1.18 [0.46,3.00]	0.73	0%	0.6
Leukopenia	3	224/218	6.52 [0.88,48.50]	0.07	39%	0.19
Lymphopenia	2	208/206	2.45 [0.76,7.82]	0.13	0%	0.48
Nausea	4	704/695	0.73 [0.30,1.73]	0.47	16%	0.31
neutropenia	4	308/301	5.31 [1.00,28.25]	0.05	69%	0.02
Rash	4	790/782	0.75 [0.46,1.20]	0.23	0%	0.47
Vomiting	3	704/695	1.05 [0.44,2.49]	0.92	0%	0.61

*P*^b^: *P*-value of *Q*-test for heterogeneity test.

### Publication bias analysis

A funnel plot and Egger's regression asymmetry test were used to access the publication bias of the literature studies. Our results showed no evidence of publications bias (PFS: *t* = −1.85, *P* = 0.137; OS: *t* = 1.39, *P* = 0.26; Supplementary Figures 1 and 2).

## DISCUSSION

In the last few years, tivantinib has been designed to treat tumors by targeting the MET. Although tivantinib has not been used in actual clinical settings, numerous clinical trials are ongoing. Results from some trials indicate that it has produced good clinical outcomes. To our knowledge, this is the first systematic review and meta-analysis that has been done to evaluate the efficacy and safety of tivantinib in solid tumor treatment. In our study, a total of 1824 patients from six trials were included, and the main tumor types in our study were NSCLC (three trials), hepatocellular carcinoma (one trial), colorectal cancer (one trial) and prostate cancer (one trial). Lung cancer was the most common malignant type, often leading to a patient's death. After ALK and EGFR, MET appears to be a potential oncogenic driver in NSCLC [18]. The most recent studies have indicated that the MET mutation was not only related to NSCLC but also contributed to the occurrence of pulmonary sarcomatoid carcinomas and lung adenocarcinomas [19, 20]. Tivantinib appears to be suitable for hepatocellular carcinoma treatment, and many clinical trials are investigating the use of this drug for treating this type of cancer [21]. So far, sorafenib was the only agent approved by the Food and Drug Administration (FDA) for the treatment of hepatocellular carcinoma, however tivantinib has shown a better effect in advanced hepatocellular carcinoma patients who have failed or are intolerant to sorafenib [22]. The mechanisms of tivantinib against hepatocellular carcinoma may be related to cell cycle G2/M phase arrest and consequent apoptosis [23]. The role of tivantinib appears to not only be limited to solid tumors as it has also been used to treat multiple myeloma [24].

According to our results, tivantinib could significantly prolong PFS, but not OS, in the overall cancer patient population. In the lung and white race subgroups, tivantinib also produced a significant improvement in PFS. However, in the high MET expression subgroup, tivantinib produced a significant improvement in OS (HR, 0.68; 95% CI, 0.48–0.95). As reported in one article, when compared with control arms, tivantinib could significant prolong OS in the high MET expression groups (HR, 0.38; 95% CI, 0.18–0.81), however, that in MET low expression groups were (HR, 1.33; 95% CI, 0.58–3.04) [15]. MET was overexpressed in many solid tumors. Tivantinib is a non-adenosine triphosphate-competitive agent that targets MET with high selectivity. It can change the structure of MET, and then block its kinase activity. Recent studies have indicated that the antitumor activity of tivantinib may not be soley due to MET inhibition [25]. However, cancer patients with high levels of MET expression or MET mutations appear to benefit from tivantinib.

Development of drug resistance is a very common problem in chemotherapy. It was also an inevitable problem in the first-line treatment of EGFR-mutated NSCLC when using EGFT-TKI inhibitors (erlotinib, gefitinib) [26]. When detected by immunohistochemistry, MET protein overexpression was found to be as high as 77% in NSCLC samples with non-squamous histology and as high as 57% in NSCLC samples with squamous cell histology [27]. Aberrant MET activation was thought to be one of the reasons for induction of drug resistance in NSCLC models [28]. In the NSCLC subgroup, three articles were included, and in all three of these articles, the treatment arms included tivantinib and erlotinib and control arms included erlotinib and placebo. According to our results, tivantinib in combination with erlotinib could significantly improve PFS. Tivantinib in combination with TKI inhibitors may provide a new strategy for the treatment of EGFR-mutated NSCLC and to some extent, may overcome NSCLC resistance to TKI inhibitors.

Tivantinib is metabolized mainly by cytochrome P450 2C19 (CYP2C19). CYP2C19 levels are very low in Caucasian populations, and about 20% of Asian populations are poor CYP2C19 metabolizers [29]. Thus, race and CYP2C19 status may be important factors that affect tivantinib's efficacy and safety. These factors should be considered when choosing tivantinib as a treatment option. According to our results, in the four studies, about 1241 of 1439 patients were white populations. Thus, we did a subgroup analysis to evaluate tivantinib's efficacy in the treatment of Caucasian populations, and tivantinib was effective in prolonging PFS in Caucasian populations.

As mentioned above, tivantinib metabolism appears to depend significantly on CYP2C19. To ensure medication safety, it has been recommended to give the proper dose of tivantinib according to CYP2C19 status. Yamamoto et al. suggested that the dose of tivantinib in combination with erlotinib should be based on CYP2C19 genotype, and in CYP2C19 extensive metabolizers, it should be 360 mg BID; in CYP2C19 poor metabolizers, 240 mg BID would be acceptable [30]. Based on this, the first phase III study was carried out. In this study, CYP2C19 extensive metabolizers received a higher dose of 360mg BID, while CYP2C19 poor metabolizers received a lower dose of 240 mg BID. However, the CYP2C19 poor metabolizers received a trend of longer PFS and OS [13]. This suggested that lower dose of tivantinib in CYP2C19 poor metabolizers was sufficient. Okusaka et al. reported that in the treatment of hepatocellular carcinoma in Japanese patients, 120 mg BID of tivantinib has been recommended regardless of CYP2C19 phenotype [31]. It has already been confirmed in phase I trials that tivantinib (360 mg BID) is well tolerated in patients with solid tumors [32]. In our study, the dose of tivantinib was 360 mg BID in all six trials, and only one trial also included a 240 mg BID arm.

In this meta-analysis, we also assessed tivantinib's safety. As reported in Scagliotti's trial, 46% of the 614 deaths were due to disease progression and 14.8% of the 142 treatment-related deaths were found in the tivantinib group [10]. Yoshioka et al. reported that tivantinib demonstrated a risk for serious interstitial lung disease and could induce death [13]. Edison et al reported that tivantinib exposure was related to ≥ grade 3 neutropenia [33]. Therefore, we should be careful about tivantinib's toxicity. According to our results, tivantinib treatment-related AEs that were ≥ grade 3 included anemia and neutropenia. This was partially consistent with previous studies.

We acknowledge that our meta-analysis had some limitations. First, as most of the included trials provided PFS and OS data, some important information such as objective response rate (ORR), progressive disease (PD), and disease control rate (DCR) were missing. Second, the sample size included in the six trials was small. Third, one of the included trials was ongoing, so that the data from this trial was not sufficient to do a proper analysis.

In summary, tivantinib did better in prolonging cancer patients’ PFS (but not OS) than some of the other drugs. Tivantinib could also significantly improve the OS in cancer patients with high levels of MET expression. A dose of 360 mg BID appears to be well tolerated.

## MATERIALS AND METHODS

### Study selection

To identify potential articles, we carried out comprehensive literature searches using PUBMED and EMBASE up to February 2017. Search terms included tivantinib or ARQ197 combined with cancer, tumor, or carcinoma. The language of publication was not limited. We also retrieved scientific meetings, unpublished trials in the clinical trial registry (http://www.clinicaltrials.gov), and relevant reviews in order to ensure the completeness and quality of the results. The included studies had to be randomized controlled trials (RCTs) and must have contained at least one clinical outcome such as PFS, overall survival (OS), and/or AEs. Case reports and single-arm and Phase I trials were excluded. In order to exclude irrelevant and overlapping studies, two investigators independently reviewed the articles.

### Data extraction

The following information was collected from all of the included RCTs: 1.) first author's surname; 2.) year of publication; 3.) number of participants; 4.) histology; 5.) trial phase; 6.) treatment arm; 7.) median age; 8.) the percentage of females; 9.) ethnicity; and 10.) the level of MET expression. The hazard ratio (HR) of the median OS and median PFS with 95% confidence intervals (CIs) were extracted to evaluate tivantinib's effects. The incidence of AEs was also retrieved in order to calculate tivantinib's safety.

### Statistical analysis

For each study, HR and 95% CI were used to assess OS and PFS between the tivantinib and control groups. We also extracted patients with AEs and total participant data from all studies and pooled them to calculate risk ratios (RRs) with 95% CI. All the data were managed by Review Manager (version 5.1, The Cochrane Collaboration, Oxford, UK) and Stata version12 (StataCorp, College Station, Texas), and a two-tailed *p* value < 0.05 was judged as statistically significant. The degree of heterogeneity and literature publication bias were measured according to methods as shown in Bao's article [34].

## SUPPLEMENTARY MATERIALS FIGURES


