# Supplementary material for: The efficacy and safety of tivantinib in the treatment of solid tumors: a systematic review and meta-analysis
Source: Oncotarget. 2017 Nov 3;8(68):113153–62. doi: 10.18632/oncotarget.22615 (PMC5762579; doi:10.18632/oncotarget.22615)
Supplement: Supplementary file 1 [file oncotarget-08-113153-s001.pdf]

# The efficacy and safety of tivantinib in the treatment of solid tumors: a systematic review and meta-analysis

## SUPPLEMENTARY MATERIALS

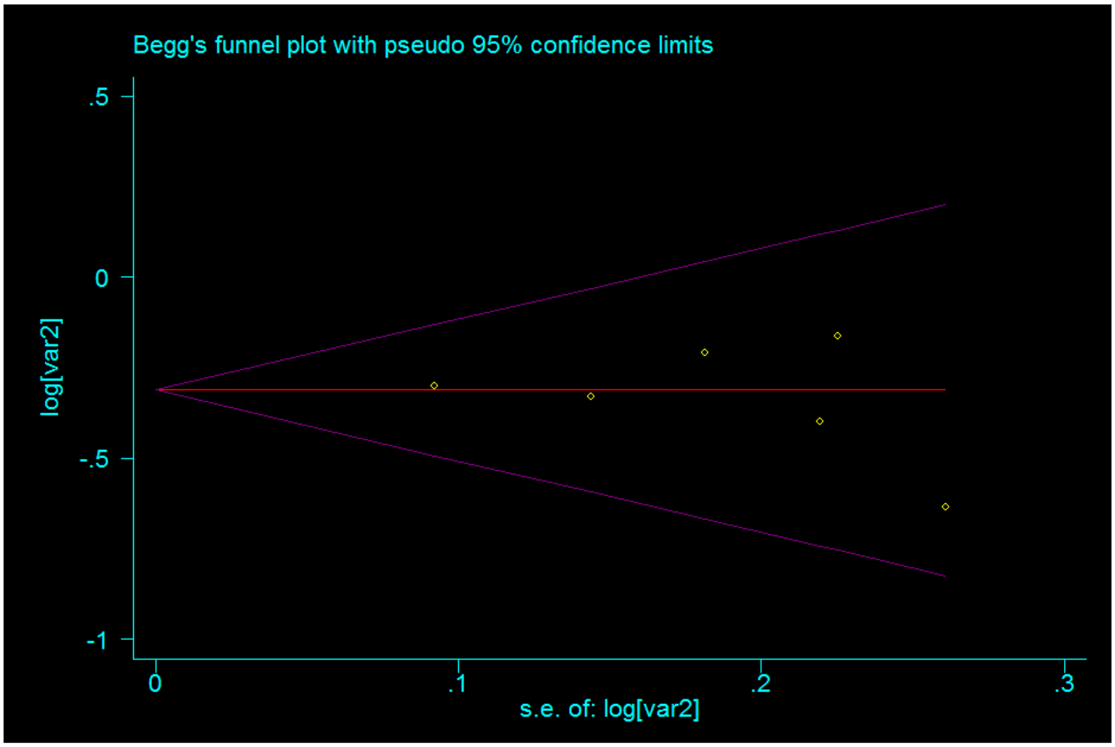


**Supplementary Figure 1: Publication bias analysis of PFS.**

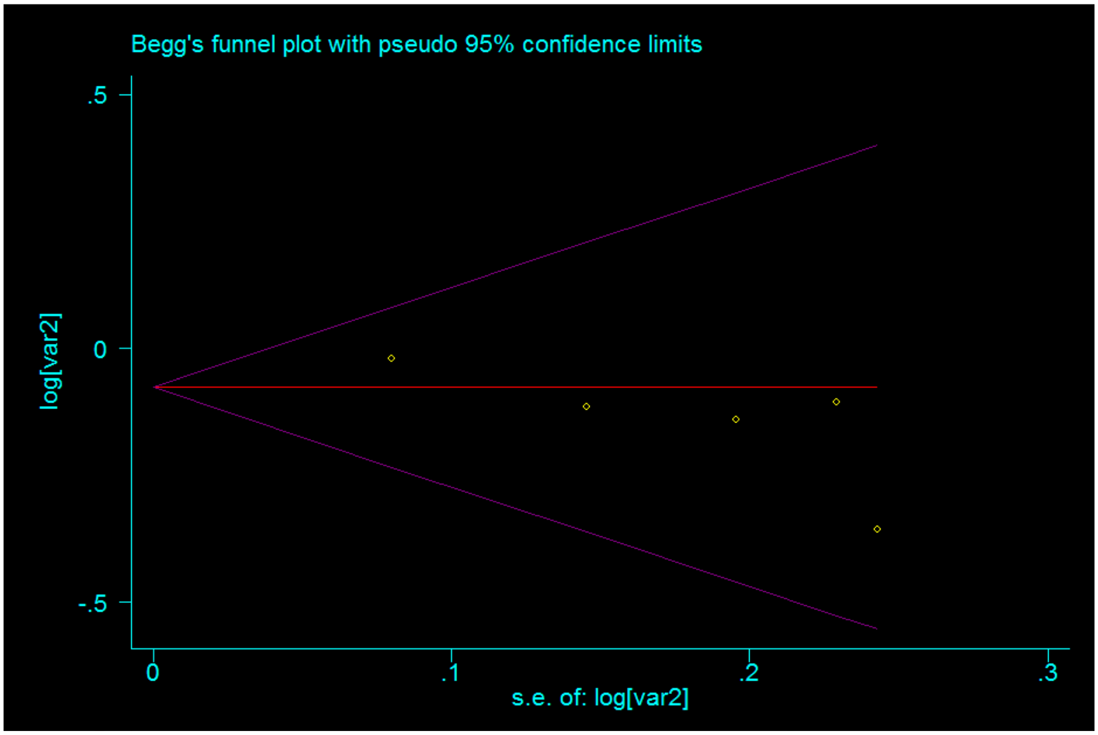


**Supplementary Figure 2: Publication bias analysis of OS.**
